# Robot-assisted bronchoscopy for pulmonary lesion diagnosis: results from the initial multicenter experience

**DOI:** 10.1186/s12890-019-1010-8

**Published:** 2019-12-11

**Authors:** Udit Chaddha, Stephen P. Kovacs, Christopher Manley, D. Kyle Hogarth, Gustavo Cumbo-Nacheli, Sivasubramanium V. Bhavani, Rohit Kumar, Manisha Shende, John P. Egan, Septimiu Murgu

**Affiliations:** 1 Division of Pulmonary, Critical Care and Sleep Medicine, Icahn School of Medicine at Mount Sinai, One Gustave L. Levy Place, Box 1232, New York, NY 10029 USA; 2 UPMC Hamot Pulmonology, UPMC Hamot, Pittsburgh, USA; 3 Section of Pulmonary Medicine, Fox Chase Cancer Center, Philadelphia, USA; 4 Section of Pulmonary and Critical Care Medicine, University of Chicago Medicine, Chicago, USA; 5 Interventional Pulmonology, Michigan State University College of Human Medicine Spectrum Health, East Lansing, USA; 6 Section of Pulmonary and Critical Care Medicine, University of Chicago Medicine, Chicago, USA; 7 Section of Pulmonary Medicine, Fox Chase Cancer Center, Philadelphia, USA; 8 Department of Cardiothoracic Surgery, UPMC Hamot, Erie, USA; 9 Interventional Pulmonology, Michigan State University College of Human Medicine Spectrum Health, East Lansing, USA

**Keywords:** Robotic bronchoscopy, Biopsy, Electromagnetic navigation, Lung cancer, Lung lesion

## Abstract

**Background:**

The Robotic Endoscopic System (Auris Health, Inc., Redwood City, CA) has the potential to overcome several limitations of contemporary guided-bronchoscopic technologies for the diagnosis of lung lesions. Our objective is to report on the initial post-marketing feasibility, safety and diagnostic yield of this technology.

**Methods:**

We retrospectively reviewed data on consecutive cases in which robot-assisted bronchoscopy was used to sample lung lesions at four centers in the US (academic and community) from June 15th, 2018 to December 15th, 2018.

**Results:**

One hundred and sixty-seven lesions in 165 patients were included in the analysis, with an average follow-up of 185 ± 55 days. The average size of target lesions was 25.0 ± 15.0 mm. Seventy-one percent were located in the peripheral third of the lung. Pneumothorax and airway bleeding occurred in 3.6 and 2.4% cases, respectively. Navigation was successful in 88.6% of cases. Tissue samples were successfully obtained in 98.8%. The diagnostic yield estimates ranged from 69.1 to 77% assuming the cases of biopsy-proven inflammation without any follow-up information (*N* = 13) were non-diagnostic and diagnostic, respectively. The yield was 81.5, 71.7 and 26.9% for concentric, eccentric and absent r-EBUS views, respectively. Diagnostic yield was not affected by lesion size, density, lobar location or centrality.

**Conclusions:**

RAB implementation in community and academic centers is safe and feasible, with an initial diagnostic yield of 69.1–77% in patients with lung lesions that require diagnostic bronchoscopy. Comparative trials with the existing bronchoscopic technologies are needed to determine cost-effectiveness of this technology.

## Background

The increasing need to efficiently and safely sample lung lesions has led to the development of virtual bronchoscopy (VB), radial endobronchial ultrasound (r-EBUS), electromagnetic navigation (EMN), fluoroscopy-based navigation, bronchoscopic trans-parenchymal nodule access (BTPNA), ultrathin bronchoscopes, and cone-beam computed tomography (CBCT) guided-bronchoscopy. The diagnostic yield for lung lesions using these modern bronchoscopic techniques continues to be suboptimal and is 40–70% [1–7].

The now commercially available robotic endoscopic system (RES; Auris Health, Inc., Redwood City, CA) has been recently FDA-approved for sampling lung lesions. In cadaveric models, robot-assisted bronchoscopy (RAB) was shown to have improved reach in the periphery of the lung in all segments when compared with 4.2 mm OD conventional thin bronchoscopes (by average generation count: 8.7 vs 5.6) [8]. This may be explained by 1) the improved structural support provided by the outer sheath, that is usually locked in the target segment (usually 3rd-4th generation) before advancing the scope, and 2) the improved ability to make subtle turns due to 4-way steering and a distal section capable of achieving articulation in pitch and/ or yaw. RAB also allows direct visualization of peripheral airways and of the biopsy tools as they are advanced outside the working channel, thereby enabling the operator to better steer the tools towards the target. The scope can be locked in position and the instruments advanced through the working channel without exertion of torque onto the bronchoscope, minimizing airway distortion. This, along with the better column strength and telescoping design, could enable higher diagnostic yields of peripheral lesions.

To date, only a small feasibility study that enrolled 15 patients using the RES was performed and showed no pneumothoraces or significant bleeding [9]. The aim of our study is to report on the initial post-marketing feasibility, safety and diagnostic yield of this technology implemented at four US-based university and community centers.

## Methods

### Patients

We retrospectively reviewed data on consecutive cases in which RAB was used to diagnose lung lesions from the very beginning of our experience with this technology (June 15th, 2018) until December 15th, 2018, at four centers in the US (University of Chicago Medical Center, Chicago, IL; University of Pittsburgh Medical Center Hamot, Erie, PA; Fox Chase Cancer Center, Philadelphia, PA; Spectrum Health, Grand Rapids, MI). The medical records of consecutive patients who were considered to require a guided bronchoscopy (EMN, VB with or without r-EBUS) and underwent RAB to diagnose lung lesions, were reviewed and included in the analysis.

### Inclusion and exclusion criteria

#### Inclusion criteria

Consecutive patients evaluated for diagnosis of lung lesions considered to require guided bronchoscopy and underwent robotic bronchoscopy (must include 1, 2, and one of 3, 4, 5 or 6):

1. 18 years of age or older
2. Acceptable candidate for an elective bronchoscopic procedure under general anesthesia
3. Pulmonary lesions suspected of being primary lung cancers identified on thin-slice CT scan, requiring bronchoscopic biopsy for diagnosis based on the guidelines [10, 11]
4. Patients with a history of lung cancer presenting with new or growing lung lesions requiring tissue diagnosis for confirming recurrence or progression of disease
5. Pulmonary lesions requiring tissue diagnosis in patients with a history of extrathoracic malignancy
6. Patients with lung lesions suspected of being due to mycobacterial or fungal infection for which a tissue diagnosis was required prior to antimicrobial therapy

#### Exclusion criteria

If inspection bronchoscopy demonstrated an endobronchial lesion that can be easily biopsied using a conventional white light bronchoscope.

### Endpoints

Device or procedure-related complications: pneumothorax (any size, even if asymptomatic), significant airway bleeding (when the robotic bronchoscope was withdrawn and a flexible bronchoscope was used for cold saline, epinephrine or endobronchial blockers), respiratory failure within 24 h of procedure (defined as new or increased requirement of supplemental oxygen or need for post-procedure ventilatory support, invasive or non-invasive).

Successful navigation: evidenced by obtaining an eccentric or concentric r-EBUS view, or diagnostic tissue on final pathology.

Diagnostic yield: Defined as the percentage of procedures yielding a diagnosis based on final pathology. If follow-up diagnostic tests confirmed a different diagnosis, or lesion growth, new lymphadenopathy or metastatic spread was detected, the procedure was considered as non-diagnostic [12]. Additionally, if the patient received treatment for lung cancer without a confirmed diagnosis or received a new diagnoses of lung cancer from any site (including from non-index lesions, or from lymph nodes by EBUS, during or after the index procedure), the procedure was considered as non-diagnostic. Diagnostic yield based on lesion characteristics (size, centrality, density, location, bronchus sign, r-EBUS view obtained) are reported considering that cases with biopsy proven inflammation for which no follow-up was available, are non-diagnostic (conservative estimates).

### Study design

#### Multi-center, retrospective, consecutive case series

##### Procedure

The Monarch Endoscopy Platform is an FDA cleared medical device (510 K #: 173760) intended to provide bronchoscopic visualization of and access to patient’s peripheral airways for diagnostic and potentially for therapeutic procedures. General anesthesia with an indwelling endotracheal tube was used for all procedures, with a tidal volume of 6–8 cc/kg and a positive end-expiratory pressure of 5–10 cm H2O. Airway inspection using a conventional white light bronchoscope was performed prior to RAB to rule out an obvious endobronchial lesion and to clear out sections from the airways. When mediastinal staging was indicated, EBUS-guided transbronchial needle aspiration (TBNA) was performed prior to RAB.

During RAB navigation, the physician uses a controller to move the robotic arms that contain rotatory pulleys to drive the bronchoscope. The bronchoscope is comprised of an outer sheath (6.0 mm) and inner scope (4.2 mm). Usually, once at a segmental bronchus, the sheath is locked in position and the scope is advanced into the smaller peripheral airways. The system uses an electromagnetic field generator and reference sensors much like other EMN bronchoscopy systems. r-EBUS was used as a confirmatory tool to verify proximity to the target. CBCT was not used in any of the cases. Biopsy tools are advanced through the working channel (2.1 mm), to biopsy the target lesion under fluoroscopy.

### Statistical analysis

Mean and standard deviation are reported for continuous variables; categorical variables are reported as percentage and counts. Associations between lesion characteristics and diagnostic yield were calculated using chi-squared tests. Multivariable logistic regression was performed to determine the odds ratio of diagnostic yield adjusted for the following characteristics: lesion location, centrality, density and size, bronchus sign and r-EBUS view. Two-tailed *p*-values of less than 0.05 were considered statistically significant for all comparisons, and analyses were performed using Stata version 14.1 (StataCorp, College Station, TX).

## Results

During the study period at the four study centers, 167 lesions were biopsied in 165 patients. Two lesions were biopsied in the same procedure in two cases. The average follow-up was 185 ± 55 days.

### Baseline and clinical characteristics of study patients

The study population consisted of 75 (46%) females. The average age at the time of the procedure was 66.5 ± 10.9 years; 77% were smokers. None of the biopsies were performed on dual anti-platelet therapy or anti-coagulants. The baseline and disease-related characteristics of the study population are presented in Table 1.

**Table 1:** Baseline and disease-related characteristics of the study patients

Female	75/165 (45.5)
Age, years	66.5 ± 10.9
< 55 years	14/165 (8.5)
55 to 65 years	51/165 (30.9)
> 65 years	100/165 (60.6)
Body mass index, kg/m^2^	28.6 ± 9.2
< 25 kg/m^2^	60/151 (40.4)
25 to 30 kg/m^2^	38/151 (25.2)
> 30 kg/m^2^	53/151 (35.1)
Family history of lung cancer	29/165 (17.6)
History of other cancers	50/165 (30.3)
History of Interstitial Lung Disease	3/165 (1.8)
History of Chronic Obstructive Pulmonary Disease	70/165 (42.4)
History of Pulmonary Hypertension	5/165 (3.0)
Smoking history	
Never	36/165 (21.8)
Former	75/165 (45.5)
Current	54/165 (32.7)
On aspirin at the time of the procedure	40/165 (24.2)

Values are means ± standard deviation or counts (%)

### Lesion characteristics

The average size of targeted lesions based on the largest measurable diameter was 25.0 ± 15.0 mm; 71.3% were ≤ 30 mm (Table 2) and 70.7% were located in the peripheral third of the lung. Bronchus-sign on the pre-procedure CT scan was observed in 106 (63.5%) lesions and 68.8% lesions were solid.

**Table 2:** Lesion characteristics

Size, mm	25.0 ± 15.0
< 10	11/167 (6.6)
10–30	108/167 (64.7)
> 30	48/167 (28.7)
Location	
Right Upper Lobe	46/167 (27.5)
Right Middle Lobe	21/167 (12.6)
Right Lower Lobe	32/167 (19.2)
Left Upper Division	40/167 (24.0)
Lingula	1/167 (0.6)
Left Lower Lobe	27/167 (16.2)
Peripheral lesion^a^	118/167 (70.7)
Lesion appearance	
Solid	125/167 (74.9)
Ground Glass	17/167 (10.2)
Mixed	15/167 (9.0)
Cavity	10/167 (6.0)

Values are means ± standard deviation or counts (%)

^a^Central lesions were defined as being located within the inner 2/3rd of the hemithorax and peripheral as those within the outer third of the hemithorax, as delineated by concentric lines around the hilum

### Procedure data

Navigation was successful in 148 (88.6%) lesions. In one case (0.6%), the RAB procedure was aborted due to a software failure. The average navigation and procedure time were 17.8 ± 19.1 min and 58.6 ± 31.4 min, respectively (this data was not available for 46 cases). The targeted lesions were detected with r-EBUS in 141 (84.4%) (eccentric view in 42.5% and concentric view in 57.5%).

### Biopsy data

Tissue samples were successfully obtained in 161 (97.6%) patients. Samples were not obtained in 4 (2.4%) cases (1 software failure, 3 unsuccessful navigation). These 4 cases were included in the analyses as failures. The overall diagnostic yield ranged from 69.1–77% assuming all the cases with biopsy proven inflammation without available follow-up (*N* = 13) were non-diagnostic and diagnostic, respectively.

The yield was 81.5, 71.7 and 26.9% for concentric, eccentric and absent r-EBUS views, respectively (*p* < 0.001). Diagnostic yield was higher for lesions with a “bronchus sign” (78.3% v 54.1%, *P* = 0.001). Yield was not different for solid versus ground glass nodules (68.8% v 70.6%, *P* = 0.74), central versus peripheral location (73.5% vs 67.8%, *p* = 0.47) and did not depend upon lesion size (45.5% for < 1 cm vs 68.5% for 1–3 cm vs 77.1% for ≥3 cm, *p* = 0.11). Outcomes based on the various nodule characteristics are shown in Tables 3 and 4. Lung adenocarcinoma accounted for 40.4% of diagnosed lesions. The pathological findings in the 114 diagnostic cases are presented in Table 5.

**Table 3:** Diagnostic yield based on lesion characteristics

	Diagnostic yield	*P*-value
Location		0.72
Right Upper Lobe	35/46 (76.1)	
Right Middle Lobe	14/21 (66.7)	
Right Lower Lobe	20/32 (62.5)	
Left Upper Division	26/40 (65.0)	
Lingula	1/1 (100)	
Left Lower Lobe	20/27 (74.1)	
Peripheral lesion	80/118 (67.8)	0.47
Bronchus sign	83/106 (78.3)	0.001
r-EBUS view		< 0.001
No view	7/26 (26.9)	
Eccentric view	43/60 (71.7)	
Concentric view	66/81 (81.5)	
Lesion endobronchial visibility	40/50 (80.0)	0.053
Lesion appearance		0.74
Solid	86/125 (68.8)	
Ground Glass	12/17 (70.6)	
Mixed	12/15 (80.0)	
Cavity	6/10 (60.0)	
Size		0.11
< 10	5/11 (45.5)	
10–30	74/108 (68.5)	
> 30	37/48 (77.1)	

Values are counts/counts (%). *P* values represent significance of association between lesion characteristic and diagnostic yield using chi-squared tests

**Table 4:** Odds ratio of diagnostic yield based on predictive characteristics

	Odds ratio (95% confidence interval)	*P*-value
Bronchus sign	2.3 (1.0–5.3)	0.04
r-EBUS view		
No view	1	–
Eccentric	7.4 (2.4–22.9)	< 0.001
Concentric	10.0 (3.2–31.1)	< 0.001

On multivariable logistic regression adjusting for the following characteristics (lesion location, centrality, endobronchial visibility, lesion appearance and size, bronchus sign and r-EBUS view), only the presence of bronchus sign and r-EBUS view were significant determinants of diagnostic yield

**Table 5:** Diagnostic findings *n* = 114

Adenocarcinoma	46 (40.4)
Small cell carcinoma	4 (3.5)
Squamous cells carcinoma	13 (11.4)
Neuroendocrine tumor	6 (5.3)
Hamartoma	2 (1.8)
Poorly differentiated lung cancer	2 (1.8)
Melanoma	1 (0.9)
Atypical cells^a^	13 (11.4)
Fungal	2 (1.8)
Appendiceal adenocarcinoma	1 (0.9)
Ovarian cancer	1 (0.9)
Non-necrotizing granuloma	3 (2.6)
Prostate cancer	3 (2.6)
Organizing pneumonia	1 (0.9)
Necrotic material^b^	2 (1.8)
Colorectal	2 (1.8)
Renal	1 (0.9)
Lymphoma	1 (0.9)
Other Benign Diagnoses^c^	10 (8.8)

Values are counts (%). In four cases tissue was not acquired due to navigation failure

^a^Atypical cells were labeled as diagnostic when they were considered sufficient to manage a nodule (with no further biopsy or follow-up required) on multi-disciplinary consensus. E.g. In a patient with head & neck cancer with lung nodules, if the lung biopsy revealed atypical cells that were considered sufficient to consider the disease as metastatic to the lung (requiring no further work-up), it was considered as a diagnostic procedure. If the finding of atypical cells required further work-up or biopsy to better characterize this, the procedure was considered non-diagnostic. E.g. A patient with suspected lung cancer, in whom a biopsy showed just atypical cells would be considered non-diagnostic

^b^Necrotic material on pathology was found in a patient whose presentation and course was consistent with a lung abscess, and in another patient with a lung lesion with newly-diagnosed histoplasmosis (on serology)

^c^These included chronic or granulomatous inflammation with or without giant cells that decreased in size on follow-up imaging

### Safety

Pneumothorax occurred in 6 (3.6%) cases, requiring chest tube placement in 4 (2.4%). Significant bleeding post-biopsies was reported in 4 (2.4%) cases. There was no need for blood transfusion, open thoracotomy or use of endobronchial blockers in any case. There were no reports of respiratory failure, deaths or any other procedure-related complications.

## Discussion

**Fig. 1:**
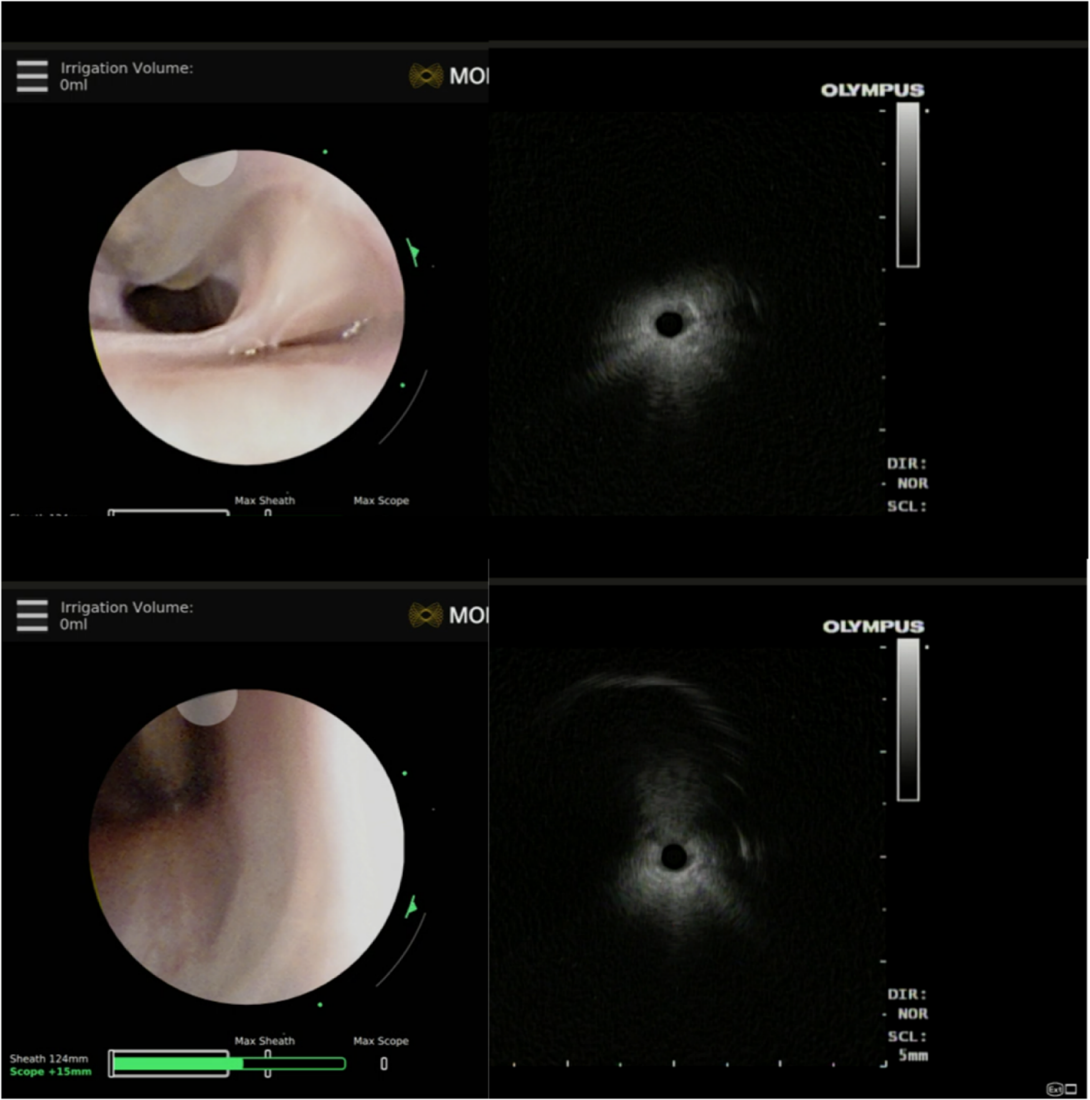
r-EBUS use to enable directional targeting of instruments. With RAB, an endoscopic view is maintained even in the smaller peripheral airways. Upper panel: the r-EBUS probe is in contact with the airway wall at the 11 o’clock position. The corresponding ultrasound image on the right shows only air artifact. Lower Panel: The r-EBUS probe is now directed to the 5 o’clock position of the airway wall and the ultrasound image reveals an eccentric view of the target lesion. An aspirating needle was oriented to penetrate the airway wall at the 5 o’clock position to obtain diagnostic tissue

This is the first study after the market release of robotic bronchoscopy in March 2018. Our study’s patients’ characteristics and average lesion size are similar to published EMN studies [12–14]. Navigation success was achieved in 88.6% with 69.1–77% overall diagnostic yield (conservative and maximum estimate). There were 13 cases in which pathology showed inflammation for which follow-up was not available. These cases were considered as non-diagnostic to provide conservative estimates of navigation success and yield based on various lesion characteristics. We provide an overall diagnostic yield range as some of these lesions could have resolved with time and have been diagnostic, if we had long-term follow-up on them [12]. We believe our definition of navigation success is meaningful for clinicians as it consists of the presence of diagnostic material on final pathology, or r-EBUS image confirmation. We did not rely only on the target image generated by the EMN software, which could be prone to multiple errors [15]. However, our definition may be subject to overestimation as it is possible that atelectasis or alveolar filling may have resulted in false positive r-EBUS images. In the absence of CBCT and confirmation of tool-in-target, it is difficult to precisely define true navigation success. In this series, diagnostic yield did not depend on lesion lobar location, centrality or size. The diagnostic yield did not depend on lesion density as well; however, we only had 17 lesions that were pure ground-glass density. The yield was better when a concentric r-EBUS view was obtained compared to an eccentric view (81.5 and 71.7%). The high yield even when an eccentric view was obtained compares favorably to previously reported rates of 48% in such cases [16]. This is likely because RAB allows stability, visualization of the point of contact of the radial probe with the airway wall and enables directional targeting of instruments [17] (Fig. 1). Biopsies were obtained in 84.6% (22/26) of cases in which r-EBUS confirmation was not obtained. The majority of these lesions (73%) were solid. The decision to biopsy was at the discretion of the operator when it was believed that the robotic scope was in the target’s proximity but in an adjacent airway based on the EM-generated target view. Our yield of 54% in the absence of bronchus sign is higher than previously reported rates of 31–44% [18, 19] but is lower than a recently published multi-center EMN study [12].

Procedure-related complications are comparable to those from other guided bronchoscopy studies, with a 3.6% pneumothorax and 2.4% bleeding rate [2, 4, 12]. While bleeding during RAB can be managed with injection of cold saline through the working channel of the robotic scope, in cases of suspected significant bleed, we have decided to disconnect the robotic scope and introduce a therapeutic flexible bronchoscope (2.8 mm working channel) to evaluate and potentially control the hemorrhage. While switching of scopes runs the risk of losing a wedged position and anatomical orientation in situations with major bleeding, we have not yet encountered such bleeding in any of our cases, likely because the robotic scope locked in a wedged position in a small peripheral airway allows containment and clotting of biopsy-related bleeding. All our cases of bleeding were controlled with just cold saline. Given that we injected cold saline prior to suctioning out the blood, we were not able to accurately quantify the volume of blood loss in each case by just looking at the return in the suction canister. There were no cases of respiratory failure or death in our study.

Our case series has several limitations. Our average follow-up period of 6 months is not sufficient to determine the true diagnostic yield and in fact large studies usually report outcomes at 12 months [2, 12]. However, as highlighted above, for the purposes of all calculations, cases that required follow-up that had not been done yet were considered as non-diagnostic. Needles and forceps were used in 100 and 96% of cases, respectively. The order in which they were used was per operators’ discretion and not captured in this analysis. In addition, the tool-specific diagnostic yield was not possible to analyze as the touch prep from forceps biopsies are reported under the cytology section and not labeled as distinct from the needle specimens. We believe that prospective studies should address the independent diagnostic yield for needles, brushes and forceps biopsies. Amongst the 71 patients diagnosed with lung cancer, adequacy of tissue obtained for genetic testing was not reported consistently in the four centers, as the practice of molecular testing for early stage lung cancer remains institution-dependent. Our definition of significant bleeding is unconventional. Unfortunately, with the robotic scope positioned in distal airway, it is very difficult to accurately assess the severity of bleeding. Future prospective studies may be able to better elucidate the true bleeding rate with RAB, without withdrawing the robotic scope. Bronchoscopy room set up, navigation and procedure times were not prospectively recorded in all cases; in the cases wherein this was documented in the medical records, the mean navigation and procedure times were 17.8 and 58.6 min, respectively. Our procedure time only reflects the robotic bronchoscopy portion of a procedure and does not include the time it took to stage the mediastinum with EBUS. Based on these data, duration of navigation and biopsy seem to be similar to other EMN-guided procedures [12].

Despite our results not demonstrating superior diagnostic yield compared to some other recent EMN-guided bronchoscopy studies [12], we believe that in the future, robotic bronchoscopy platforms may eventually enable operators to more precisely navigate to the periphery of the lung and potentially allow for bronchoscopic therapeutic ablation of malignant lesions. As of now, in our opinion, when available, robotic bronchoscopy should be offered to all patients with suspicious peripheral lung lesions that also require 1) concurrent guidelines-recommended EBUS-TBNA lymph node staging for CT-PET normal mediastinum or prior to SBRT; or 2) preoperative tissue diagnosis based on questionable operability, patient or surgeon’s preference.

## Conclusion

The results of this analysis suggest that in patients with lung lesions requiring biopsy, post-marketing RAB implementation in community and academic centers is safe with initial diagnostic yield and complication rates similar to existing technologies. Long-term follow-up is required to better establish the true diagnostic yield and delineate the factors affecting it. Comparative trials with existing guided bronchoscopy platforms are warranted for determining cost-effectiveness of this technology in diagnosing lung nodules.

## Data Availability

The datasets used and analysed during the current study are available from the corresponding author on reasonable request.

## Abbreviations

ACCP: American College of Chest Physicians
BTPNA: Bronchoscopic Transparenchymal Nodule Access
CBCT: Cone beam computed tomography
CT: Computed tomorgraphy
EBUS: Endobronchial ultrasound
EMN: Electromagnetic navigation
NCCN: National Comprehensive Cancer Network
OD: Outer diameter
PET: Positron emission tomography
POE: Point of entry
RAB: Robot-assisted bronchoscopy
r-EBUS: Radial endobronchial ultrasound
RES: Robotic endoscopic system
TBNA: Transbronchial needle aspiration
VB: Virtual bronchoscopy
